# Global Financing and Long-Term Technical Assistance for Multidrug-Resistant Tuberculosis: Scaling Up Access to Treatment

**DOI:** 10.1371/journal.pmed.1001738

**Published:** 2014-09-30

**Authors:** Thomas J. Hwang, Salmaan Keshavjee

**Affiliations:** 1 Faculty of Arts and Sciences, Harvard University, Cambridge, Massachusetts, United States of America; 2 The Blackstone Group, London, United Kingdom; 3 Program in Infectious Disease and Social Change, Department of Global Health and Social Medicine, Harvard Medical School, Boston, Massachusetts, United States of America; 4 Partners in Health, Boston, Massachusetts, United States of America

## Abstract

Thomas Hwang and Salmaan Keshavjee argue that a market-based strategy combined with long-term in-country technical assistance should be used to scale-up access to the treatment of multi-drug resistant tuberculosis

*Please see later in the article for the Editors' Summary*

**Summary Points**

- Multidrug-resistant tuberculosis (MDR-TB) is a leading public health concern, particularly in low- and middle-income countries, necessitating coordinated international action to prevent its spread and effectively treat the infected.
- The cost of treatment for MDR-TB is over 200 times the comparable cost for a drug-susceptible tuberculosis (TB) patient. Data show that prices for three of the currently most expensive drugs have increased dramatically since 2001, outpacing inflation.
- Many of the high MDR-TB burden countries were ranked by WHO as being in the bottom 50% of health systems worldwide. Without sufficient technical, human, and organizational resources, weak health systems can pose a significant barrier to access to treatment.
- In order to achieve the goal of eradicating MDR-TB, policymakers should implement a two-pronged intervention that pools donor resources for the coupling of market-oriented solutions to MDR-TB drug prices and targeted investments in health systems strengthening and innovative care delivery models. Innovative policy mechanisms piloted for other infectious diseases, such as pneumococcal vaccine, may offer lessons for the MDR-TB context.

Multidrug-resistant tuberculosis (MDR-TB), strains of *Mycobacterium tuberculosis* resistant to the first-line antituberculosis drugs rifampicin and isoniazid, is a global public health concern and remains a leading cause of mortality and morbidity in low- and middle-income countries [1]. In response, World Health Organization (WHO) member states have called for action to “achieve universal access to diagnosis and treatment of multidrug-resistant and extensively drug-resistant tuberculosis” [2]. However, the drive to eradicate MDR-TB using current strategies remains an uphill battle [3],[4]. Extensively drug-resistant tuberculosis (XDR-TB), a form of MDR-TB with additional resistance to the backbone of the second-line antituberculosis regimen—fluoroquinolones and injectable agents—has been officially reported in 92 countries [5].

Making MDR-TB treatment more affordable would be an important step towards improving patient access to care and reducing the significant public health burdens arising from the spread of this highly contagious airborne disease [6],[7]. Here, we assess changes in MDR-TB drug prices since 2001 and identify limitations in the current system, focusing on the barriers to care delivery posed by high costs of treatment and weak health systems. Using pneumococcal vaccine as a case study to inform the fight against the global tuberculosis (TB) pandemic, we argue that a market-based strategy coupled with long-term in-country technical assistance should be utilised to scale up access to MDR-TB treatment.

## Current Landscape of MDR-TB Drug Market

The high price of medicines can be a significant barrier to access to treatment, particularly in resource-limited settings [8],[9]. In 2000–2001, key stakeholders launched the Green Light Committee Initiative (GLC) and Global Drug Facility (GDF) to promote access to and rational use of concessionally priced and quality-assured TB drugs [10],[11]. The linked GLC-GDF mechanism has been successful in reducing prices for first-line drugs and for second-line drugs [12]. As a result of these reforms, the average price of second-line drugs in low-income countries fell by 38%–92% compared to prices before these reforms. However, shortages and delays of quality-assured MDR-TB drugs have since become a recurring problem, notably of para-aminosalicylate sodium (PAS) in 2006 and capreomycin in 2007 and 2011 [13].

**Figure 1:**
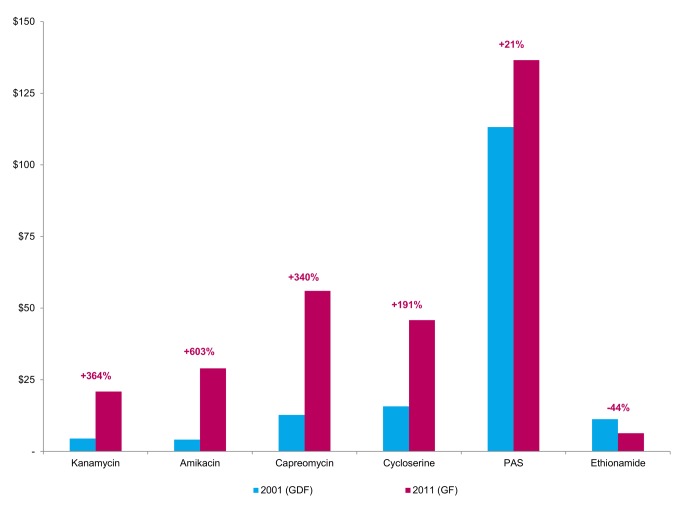
Prices for a month's treatment of selected second-line agents in 2001 and 2011. Prices are given in constant 2011 dollars. Percentages indicate changes between highest actual and lowest quoted prices. GDF = Global Drug Facility. GF = Global Fund grant recipients.

**Figure 2:**
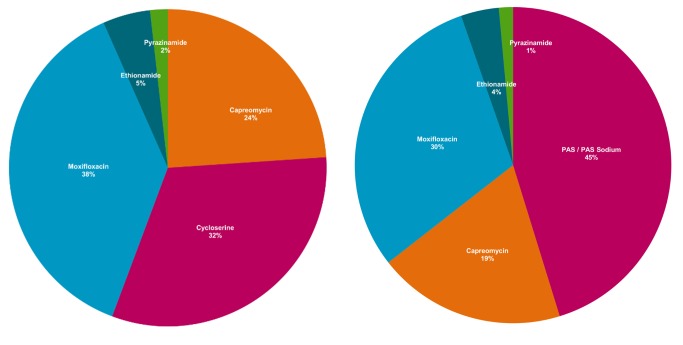
WHO recommended MDR-TB regimens, by contribution of mean cost of drug. Authors' analysis of Doctors without Borders (MSF) data (see [14]). Proportions are calculated relative to the total cost of the regimen, and may not sum to 100 due to rounding.

Data from WHO and the Global Fund to Fight AIDS, Tuberculosis, and Malaria (“Global Fund”) show that prices for three of the currently most expensive drugs—PAS, cycloserine, and capreomycin—have increased significantly since 2001 (Figures 1 and 2). Consequently, for an MDR-TB patient, the nominal cost of second-line antituberculosis medicines can be over 200 times greater than the comparable cost for a drug-susceptible TB patient [14]. While most low-income, high-burden countries receive funding for their MDR-TB treatment programmes from the Global Fund, thereby allowing these countries to offer medicines at no cost to patients, the country-level organization and delivery costs can represent a substantial proportion of the total budget for national TB programmes, and may thus still hinder country-driven scale-up of treatment [15]. Because many of these same medicines are associated with serious adverse events, their use necessitates the additional costs of close laboratory and clinical follow-up [16],[17].

These increases in second-line drug prices—despite substantial price reductions seen as part of the 2000–2001 reforms—highlight the complexity of maintaining affordable prices in the MDR-TB market. One explanation may be the small and, in some cases, declining number of quality-assured suppliers of second-line drugs [13],[18]. Equally, market-based solutions must be tied to delivery interventions to ensure that infected patients are adequately identified and treated in sufficient scale to offset price reductions. Without meaningful competition in a market already characterised by low volumes and high manufacturing costs, central authorities will likely find it increasingly difficult to use negotiation alone to sustain reduced prices for MDR-TB drugs.

## Market-Shaping Strategies in Other Disease Contexts: Case Study of Pneumococcal Vaccine

As with MDR-TB, concerns over the cost of treatment and burden of disease plagued pneumococcal vaccine efforts. An “advanced market commitment” (AMC) was proposed in 2005 to encourage market entry and rapid scale-up of pneumococcal vaccine use in low- and middle-income countries [19]. Under this programme, participating manufacturers are subsidised proportionately to their supply commitment, with a high price guaranteed for the first two years to incentivise investments in manufacturing capacity and research and development. For the remaining years of the programme, participating suppliers are paid a lower “tail price” to cover the marginal costs of production, of which Global Alliance for Vaccines and Immunization (GAVI)-supported countries pay a copayment of $0.20 or more per dose, depending on their national income; the remainder is paid by GAVI funds [20]. In return, manufacturers commit to supply actual demand or their committed supply level, whichever is lower.

In 2007, a consortium of donors, including the Bill and Melinda Gates Foundation, pledged a total of $1.5 billion for a goal of 200 million doses of pneumococcal vaccine annually by 2015. In the first three years of the programme, the number of high-income countries with the second-generation pneumococcal vaccine was roughly twice that of low-income countries, compared with a ratio of nearly 9 to 1 for the first generation version [21]. However, the programme has faced a number of challenges. GAVI has acknowledged that it will not be able to meet demand for some participating countries due to shortfalls in short-term supply availability. Some have also questioned whether GAVI had negotiated sufficiently on price [22].

Nonetheless, the case study of pneumococcal disease illuminates best practices in ensuring access to essential medicines and vaccines, whilst also incentivising market entry and manufacturer investments in research and development (R&D). However, there are a number of important differences between the pneumococcal vaccine case and MDR-TB treatment. First, there are limited incentives for manufacturer interest in the MDR-TB market, which is characterised by low volumes, high disease burden in poor countries, and highly variable demand. Second, unlike vaccination-based health interventions, MDR-TB treatment typically involves greater time, more resources, and higher organizational and delivery investments, particularly relating to the duration of treatment monitoring.

## Health Systems and Challenges to Care Delivery

In addition to the costs of treatment, it has become clear in recent years that “demand side” factors also play an important role in defining access to MDR-TB treatment and the size of the market for second-line antituberculosis drugs. Data from the Global Fund show that some high-burden countries have returned monies because they do not have the capacity to absorb it. For example, Nigeria and Indonesia are two lower-middle–income countries that have received substantial funding from the Global Fund for their national TB programmes. Yet, between 2010 and 2012, Nigeria and Indonesia had drawn only 60% and 50%, respectively, of the TB-specific funds allocated to it by the Global Fund [23]. In fact, the Global Fund reports suggested that the reason allocated funds were not used was because of major delays in carrying out programme activities such as the signing of grant contracts with sub-recipients, launch of media campaigns, and procurement of diagnostic materials.

Indeed, a closer look at the high MDR-TB burden countries reveals that the vast majority (21 out of 27, 78%) were ranked by the WHO as being in the bottom 50% of health systems worldwide (Table 1). Without sufficient technical, human, and organizational resources, weak health systems can pose a significant barrier to access to treatment. In several countries, the integration of tuberculosis services into primary care and HIV interventions remains lacking, which is troubling since poor access to healthcare can lead to low treatment success [24]. In addition, a focus on hospital-based treatment may severely limit treatment access. Emphasising inpatient treatment can alienate patients, who must sacrifice up to two years of forgone productivity because of hospitalisation, while also placing further strains on hospital systems and the health workforce [25].

**Table 1:** Disease burden and health and economic characteristics of the 27 high-burden MDR-TB countries.

Country	Est. number of MDR-TB patients ('000)	% est. MDR-TB cases enrolled on treatment	Health system overall performance ranking	Public spending on health	Living on <$1.25/day
			(N = 191; 1997)	(% of GDP)	(2002–2011)
***Africa***					
D.R. Congo	2,900	6%	188	3.4	87.7
Ethiopia	2,100	14%	180	2.6	39.0
Nigeria	3,600	3%	187	1.9	68.0
South Africa	8,100	80%	175	3.9	13.8
***Europe and Eastern Mediterranean***					
Armenia	250	40%	104	1.8	<1
Azerbaijan	2,800	15%	109	1.2	<1
Belarus	2,200	>90%	72	4.4	<1
Bulgaria	100	36%	102	3.7	<1
Estonia	70	77%	77	4.7	<1
Georgia	630	>90%	114	2.4	15.3
Kazakhstan	8,800	82%	64	2.5	<1
Kyrgyzstan	1,800	27%	151	3.5	6.2
Latvia	120	86%	105	4.1	<1
Lithuania	300	>90%	73	5.2	<1
Pakistan	11,000	10%	122	0.8	21.0
Rep. of Moldova	1,700	50%	101	5.4	<1
Russia	46,000	40%	130	3.2	<1
Tajikistan	910	59%	154	1.6	6.6
Ukraine	6,800	>90%	79	4.4	<1
Uzbekistan	4,000	37%	117	2.8	–
***Southeast Asia and Western Pacific***					
Bangladesh	4,200	12%	88	1.2	43.3
China	59,000	3%	144	2.7	13.1
India	64,000	22%	112	1.2	32.7
Indonesia	6,900	6%	92	1.3	18.1
Myanmar	6,000	7%	190	0.2	–
Philippines	13,000	15%	60	1.3	18.4
Vietnam	3,800	19%	160	2.6	40.1

MDR-TB prevalence and treatment enrolment statistics are from the 2013 Global TB Report by the World Health Organization [5] and represent notified cases of TB. The total burden of MDR-TB may be higher than these estimates. Health systems rankings are from the WHO's World Health Report 2010, Annex, table 10. The systems ranking was discontinued by the WHO in 2000 and has not been updated since then. Poverty statistics are from the 2013 Human Development Report by the United Nations Development Programme.

## Recommendations

The persistently high cost of a full 24-month course of chemotherapy raises important questions about international efforts to ensure affordable MDR-TB care. Moreover, weak health systems are present in many of the high-burden countries, meaning that market interventions focused on price alone are unlikely to address the root causes of global deficits in MDR-TB treatment and control. Hence, charting a new course forward for the global movement to eradicate drug-resistant TB should follow a two-pronged approach: pooling donor resources for the coupling of market-oriented solutions to MDR-TB drug prices and targeted investments in health systems strengthening and innovative care delivery models.

A coordinated, evidence-based public health intervention for MDR-TB could include the following components (Box 1).

**Box 1. A Global Intervention for MDR-TB Treatment Access**

1. **Global strategic stockpile.** A strategic stockpile could reduce short-term demand variability, prevent shortages of second-line drugs, and strengthen negotiations with manufacturers.
2. **Advance purchase commitments.** A high-profile commitment could ensure that all patients can access safe and effective treatments entering the market, whilst also potentially stimulating investments in research and development for new treatments for MDR-TB.
3. **Technical assistance.** Novel models of technical assistance that build in-country health systems capacity are essential to not only sustain any price reductions but also reliably diagnose infected patients and deliver care throughout their treatment.

### Global strategic stockpile of second-line drugs

A global strategic reserve supply (or stockpile) of second-line drugs could reduce short-term demand variability and prevent shortages of second-line drugs. In December 2013, UNITAID announced $15 million in funding to expand the Strategic Rotating Stockpile (SRS) for MDR-TB medicines. However, the scale of the existing SRS remains limited, covering an estimated 5,800 MDR-TB treatments [26]. The planned transition of the SRS to the Global Fund by the end of 2014 presents an opportunity for the international community to evaluate ways that the strategic stockpile could address some of the market challenges described herein.

Although it currently acts as a purchaser of last resort, the SRS could play a greater role in negotiations with manufacturers of second-line drugs. For example, the Global Fund and international partners could commit to supplying the SRS with a fixed percentage of all drug volumes procured by GDF, thereby “topping up” orders made through the GLC-GDF mechanism. While the implementation of a pooled purchasing strategy would need to be carefully defined, GDF and the SRS could look to the example of the Clinton's Health Access Initiative and the United States President's Emergency Plan for AIDS Relief, which have successfully used their position as major purchasers of a wide range of medicines on behalf of developing countries to not only negotiate favourable prices for their beneficiaries but also meaningfully reduce demand-side uncertainty for manufacturers [27].

### Advance purchase commitments and parallel review

Given the prospect of the first new treatment agents for MDR-TB in decades, an expanded SRS mechanism could also be utilised by donors to pilot an advance purchase commitment for MDR-TB, modelled after the AMC for pneumococcal vaccine. For example, the US Food and Drug Administration (FDA) recently approved bedaquiline (Sirturo), a novel diarylquinoline, for the treatment of MDR-TB [28]. Similarly, in November 2013, the European Medicines Agency (EMA) approved delamanid (Deltyba), a nitro-dihydro-imidazooxazole derivative, for treatment of MDR-TB patients without other treatment options because of resistance or tolerability [29]. To prevent shortages associated with the introduction of new technologies in resource-limited settings, in coordination with other donors, the SRS could commit to purchasing sufficient doses of a new drug to treat identified MDR-TB patients. Drawing on their expertise, the Global Fund and its partners, including nongovernmental and community organisations, could also coordinate with manufacturers to streamline the delivery of new medicines to patients and providers in high-burden TB countries. In turn, these commitments could further stimulate investments in R&D for new treatments for MDR-TB by guaranteeing a market for safe and effective products [30].

In addition, the WHO and Global Fund could explore the creation of a parallel review process, in which WHO review occurs simultaneously with negotiations for these advance purchase commitments. One precedent for such a process arose in the US in 2011 when the FDA and the Centres for Medicare and Medicaid Services, a large public payer, launched a pilot programme for the parallel review of medical devices for approval and coverage [31].

### Novel models of technical assistance

It is critical that the above interventions occur in parallel to address health system barriers to MDR-TB treatment [3],[4], specifically building systems capable of diagnosing infected patients, rapidly initiating treatment, and reliably delivering care throughout their treatment. Despite the provision of short-term technical assistance through various global mechanisms, many countries remain unable to mount the type of health intervention required to treat MDR-TB at scale, as demonstrated by the Nigeria and Indonesia examples. In 2008, the US Institute of Medicine recognized the challenge in expanding access to treatment of MDR-TB without building enduring in-country capacity [32]. Long-term on-site technical assistance should draw on the experience of successful regional treatment programmes and would be particularly meaningful for countries seeking to augment capacity in performing mycobacterial culture, drug susceptibility testing, and rapid molecular genetic tests for MDR-TB [32].

## Conclusion

Achieving the three goals of access, affordability, and sustainability in the MDR-TB context is a public health imperative. Novel financing mechanisms, such as advance purchase agreements and a global strategic stockpile, are necessary to improve the affordability of and access to MDR-TB treatment—in turn, improving the efficiency of use of country and donor resources. In parallel, any novel financing mechanism must also address the implementation gap affecting MDR-TB scale-up, such as through targeted investments in health systems strengthening and innovative care delivery models.

## Abbreviations

AMC: advanced market commitment
EMA: European Medicines Agency
FDA: United States Food and Drug Administration
GAVI: Global Alliance for Vaccines and Immunization
GDF: Global Drug Facility
GLC: Green Light Committee Initiative
MDR-TB: multidrug-resistant tuberculosis
PAS: para-aminosalicylate sodium
R&D: research and development
SRS: Strategic Rotating Stockpile
TB: tuberculosis
XDR-TB: extensively drug-resistant tuberculosis
